# How do colorectal cancer patients rate their GP: a mixed methods study

**DOI:** 10.1186/s12875-021-01427-7

**Published:** 2021-04-08

**Authors:** Tania Blackmore, Lynne Chepulis, Rawiri Keenan, Jacquie Kidd, Tim Stokes, David Weller, Jon Emery, Ross Lawrenson

**Affiliations:** 1 Medical Research Centre, University of Waikato, Hamilton, New Zealand; 2 Auckland University of Technology, Auckland, New Zealand; 3 Department of General Practice and Rural Health, Dunedin, University of Otago, Dunedin, New Zealand; 4 Centre for Population Health Studies, The University of Edinburgh, Edinburgh, Scotland, UK; 5 Medicine, Dentistry and Health Sciences, The University of Melbourne, Melbourne, Australia

## Abstract

**Background:**

New Zealand (NZ) has a high incidence of colorectal cancer (CRC) and low rates of early diagnosis. With screening not yet nationwide, the majority of CRC is diagnosed through general practice. A good patient-general practitioner (GP) relationship can facilitate prompt diagnosis, but when there is a breakdown in this relationship, delays can occur. Delayed diagnosis of CRC in NZ receives a disproportionally high number of complaints directed against GPs, suggesting deficits in the patient-GP connection. We aimed to investigate patient-reported confidence and ratings of their GP following the diagnostic process.

**Methods:**

This study is a mixed methods analysis of responses to a structured questionnaire and free text comments from patients newly diagnosed with CRC in the Midland region of NZ. A total of 195 patients responded to the structured questionnaire, and 113 patients provided additional free text comments. Descriptive statistics were used to describe the study population and chi square analysis determined the statistical significance of factors possibly linked to delay. Free text comments were analysed using a thematic framework.

**Results:**

Most participants rated their GP as ‘Very good/Good’ at communication with patients about their health conditions and involving them in decisions about their care, and 6.7% of participants rated their overall level of confidence and trust in their GP as ‘Not at all’. Age, gender, ethnicity and a longer diagnostic interval were associated with lower confidence and trust. Free text comments were grouped in to three themes: 1. GP Interpersonal skills; (communication, listening, taking patient symptoms seriously), 2. Technical competence; (speed of referral, misdiagnoses, lack of physical examination), and 3. Organisation of general practice care; (appointment length, getting an appointment, continuity of care).

**Conclusions:**

Māori, females, and younger participants were more likely to report low confidence and trust in their GP. Participants associate a poor diagnostic experience with deficits in the interpersonal and technical skills of their GP, and health system factors within general practice. Short appointment times, access to appointments and poor GP continuity are important components of how patients assess their experience and are particularly important to ensure equal access for Māori patients.

## Background

Trust and confidence in general practitioners (GPs) is usually reported as high [1]. Factors associated with patient confidence in GPs include clear explanations of tests and treatments, involving patients in decisions about care and patient perceptions that their symptoms are being taken seriously. When trust breaks down and care is perceived to be sub-optimal, conflict can ensue. In New Zealand (NZ) any complaint about health practitioners can be referred to the Health and Disability Commissioner (HDC). A report for the HDC (2004–2013) indicated that approximately 10% of complaints about GPs involved a perceived delay in diagnosis of cancer [2]. Colorectal cancer (CRC) - the second most common cancer in NZ [3] - was over-represented, comprising 27% of these complaints. The nature of complaints highlighted in the report were a lack of clinical examinations, patient perceptions of inadequate follow-up of symptoms and poor GP communication.

Based on international comparisons, NZ has a low rate of early stage CRC [4], with fewer than 12% of patients diagnosed at stage I [5]. While not yet having a fully implemented national bowel cancer screening program is a contributing factor, CRC is also difficult to diagnose [6], with a complex diagnostic process for both patients and GPs. For patients, symptoms can be nonspecific and difficult to recognise as potentially serious in nature and in need of medical investigation [7–9]. For GPs, the difficulty lies in the frequency of bowel symptoms in primary care and interpretive complications. Non-cancer diagnoses are much more common, and can include conditions such as irritable bowel syndrome, inflammatory bowel disease (Crohn’s and ulcerative colitis) and diverticular disease. There is accordingly, significant potential for misdiagnosis, especially in the presence of comorbidity [10, 11] or existing gastro-intestinal issues that can confound the presence of CRC symptoms [7, 12, 13].

The patient-GP relationship is an integral aspect of the diagnostic process. A GPs interpersonal skills (e.g., listening, empathy, being non-judgemental) and technical competence (e.g., knowledge, performing physical examinations, proactively investigating, following up on referrals) can either facilitate or impede prompt diagnosis. Good GP communication helps patients feel connected to their GP and the care provided [14], but a lack of empathy, inattentive listening and not taking patients seriously can lead to negative patient-GP interactions [15], patient dissatisfaction [9] and complaints [16]. Technical competence is also an important consideration in the patient-GP relationship, but can be outweighed by interpersonal competence [17], highlighting the importance patients place on a GPs’ personal style during interactions.

Given the prevalence of CRC complaints in primary care in NZ, it is important to investigate patient reported confidence in their GP following the diagnostic process. We therefore interviewed patients recently diagnosed with CRC using a structured questionnaire to investigate factors that lead to high (or low) patient ratings of trust and confidence in their GP and how these factors contribute to the overall diagnostic experience.

## Method

### Participants

Participants were selected from the Midland region, which includes Waikato (population: 400,000+), Tairawhiti (population: 40,000+) and Lakes (population: 100,000+) District Health Boards (DHBs). Participants were recruited as part of a larger prospective study where data were collected via researcher-assisted interviews that administered a 52-item questionnaire based on the SYMPTOM questionnaire [12]. Questionnaire data was then analysed based on the Model of Pathways to Treatment (MPT) [18] framework. Initial recruitment occurred through referral from a CRC cancer nurse specialist (CNS) at each of these DHBs and participants were contacted to arrange a time and day for interview. Additional recruitment within the Waikato region took place via mail out of study information from patient lists obtained from Waikato DHB, use of a poster placed at Waikato hospital and in private consulting rooms, and a Bowel Cancer NZ social media page. No interviews took place until a signed consent form or written consent via email or text message was obtained.

Participants were selected for recruitment if they had been diagnosed and interviewed within 12 months of diagnosis (study period from 2016 to 2019) and had not participated in bowel screening (where screening has been implemented regionally (e.g., Lakes DHB)). Interviews took place from April 2018 to March 2020. During the interview, participants were invited to speak about their experience of being diagnosed with CRC, with a particular focus on patient-reported symptoms and the timeline from symptom onset to when a health care professional (usually a GP) was consulted. The results of this larger study are not reported here. The current study reports on Section 3 of the questionnaire, which asks about health service utilisation and the patient-GP experience using three key questions (see Tables 2, 3 and 4).

Responses to these key questions were collected using a 5 –point Likert rating scale ranging from ‘Very good’ to ‘Very poor’ or ‘Yes definitely’ to ‘Not at all’. All three questions also included ‘Doesn’t apply’ and ‘Don’t know’ as possible response options. In addition, free text comments were recorded verbatim by the researcher at any point during the interview, but were specifically prompted in Section 3 due to these questions’ particular relation to the patient-GP experience. Ethical approval for this study was granted by the New Zealand Health and Disability Ethics Committee (Ref: 17/NTB/156).

### Delay intervals

The MPT [18] provided the theoretical framework for data analysis and defines four intervals from first symptom/bodily change to commencement of treatment (appraisal, help seeking, diagnostic, and pre-treatment). This study reports only on the total diagnostic interval (TDI), defined as the date of first symptom onset to date of diagnosis as guided by the Aarhus statement [19] and a previous study [12]. Diagnostic intervals were defined as > 120 days or < 120 days, based on Australian clinical guidelines [20].

### Data analysis

Descriptive statistics were used to describe the study population and the characteristics of the patients who provided free text comments. Chi square analysis was used to determine any statistical significance. All tests for significance were two-tailed with *p* < 0.05 considered a statistically significant result. All data analyses were performed using SPSS version 25 (New York, US). Additional free text comments were compiled, and these responses were analysed by the primary author (TB). As described in a similar study [11], free-text comments in the current study were also considered as unstructured and unguided qualitative data. Using an inductive approach guided by the data, thematic analysis techniques [21] were applied whereby comments were coded manually using highlighters in an Excel spreadsheet and then analysed for themes.

## Results

Table 1 shows the characteristics of the current cohort. The participants were mostly aged 70–79 (33.8%), non-Māori (84.6%), male (55.9%) and had been diagnosed through their GP (64.1%). Over half (53.3%) of participants had a TDI of more than > 120 days. Dates needed to calculate TDI were unknown for 12 participants. Seventy-three (37.4%) participants could not get an appointment with a GP or nurse within 24 h of calling their medical practice for the purpose of making an appointment (for any reason). The main reason why an appointment could not be made was a lack of available appointments (24.6%). From the current sample, 113 (57.9%) participants offered free text comments relevant to GP-related care (characteristics are shown in the right hand panel of Table 1).

**Table 1:** Characteristics of the whole study population (*N* = 195) (left) and characteristics of participants who offered additional free text comments (*n* = 113) (right)

Factors	Whole cohort		Free text comments	
	***N*** = 195	%	***n*** = 113	%
**Age**				
< 40	4	2.1	3	2.7
40–49	15	7.7	11	9.7
50–59	30	15.4	20	17.7
60–69	47	24.1	27	23.9
70–79	66	33.8	40	35.4
80+	33	16.9	12	10.6
Ethnicity				
non-Māori	165	84.6	96	85.0
Māori	29	14.9	17	15.0
Unknown	1	0.5	0	0.0
**Gender**				
Male	109	55.9	58	51.3
Female	86	44.1	55	48.7
**Mode of diagnosis**				
Through a GP	125	64.1	76	67.3
Incidental finding	29	14.9	19	16.8
Presentation to ED	29	14.9	14	12.4
Other	12	6.2	4	3.5
**Total diagnostic interval**				
< 120 days	79	40.5	43	38.1
> 120 days	104	53.3	67	59.3
Unknown	12	6.2	3	2.7
**Comorbidities**				
0–1	156	80.0	94	83.2
2+	39	20.0	19	16.8
**In the past 12 months, were you able to get an appointment within 24 h?**				
Yes	73	37.4	57	50.4
No	120	61.5	55	48.7
Don’t know	2	1.0	1	0.9
**Why couldn’t you get an appointment?**				
There were no appointments	48	24.6	34	30.1
GP I did not want to see	12	6.2	11	9.7
No appointments/ GP I did not want to see	8	4.1	6	5.3
Can’t always see the same GP/GP unavailable	5	2.6	2	1.8
Another reason	2	1.0	4	3.5

Tables 2, 3 and 4 show how participants responded to three key questions relevant to the patient-GP relationship. Eighteen participants (9.2%) rated their GPs communication as ‘Neither good nor bad/Poor or Very poor’. The majority (79.5%) of participants (*n* = 155) rated their GP involving them in decisions about their care as ‘Very good/Good’. When asked for an overall judgment of confidence and trust in their GP, 40 participants (20.5%) rated that level of confidence and trust as ‘Yes, to some extent/Not at all’. Chi-square analysis showed that age (*p* = 0.004) and gender (*p* = 0.028) were significantly associated with the confidence and trust rating. Proportionally, more Māori participants gave a ‘Yes, to some extent/Not at all’ rating of overall confidence and trust in their GP compared to non-Māori (37.9% (11/29) vs. 17.6% (29/165)) but this did not reach statistical significance (*p* = 0.738).

**Table 2:** Participant responses to the question: thinking about your last visit to a GP, how good was the doctor at explaining your health conditions and treatments in a way that you could understand?

Factors	Very good/Good		Neither good nor bad/poor or very poor		Doesn’t apply	Totals		
	*n* = 175	%	*n* = 18	%	*n* = 2	*N* = 195	%	*p*
**TDI**								
< 120 days	73	92.4	5	6.3	1	79	40.5	0.919
> 120 days	90	86.5	13	12.5	1	104	53.3	
Unknown	12	100.0	0	0.0	0	12	6.2	
**Age**								
< 60	42	85.7	6	12.2	1	49	25.1	0.270
60+	133	91.1	12	8.2	1	146	74.9	
**Gender**								
Male	97	89.0	10	9.2	2	109	55.9	0.404
Female	78	90.7	8	9.3	0	86	44.1	
**Ethnicity**								
non-Māori	148	89.7	16	9.7	1	165	84.6	0.883
Māori	26	89.7	2	6.9	1	29	14.9	
Unknown	1	100.0	0	0.0	0	1	0.5	
**Comorbidity**								
0–1	139	89.1	15	9.6	2	156	80.0	0.071
2+	36	92.3	3	7.7	0	39	20.0

**Table 3:** Participant responses to the question: How good was the doctor at involving you in decisions about your care, e.g. discussing different treatment options?

Factors	Very good/good		Neither good nor bad/poor or very poor		Doesn’t apply	Totals		
	*n* = 155	%	*n* = 24	%	*n* = 16	*N* = 195	%	*p*
**TDI**								
<120 days	69	87.3	4	5.1	6	79	40.5	0.168
>120 days	75	72.1	20	19.2	9	104	53.3	
Unknown	11	91.7	0	0.0	1	12	6.2	
**Age**								
< 60	38	77.6	7	14.3	4	49	25.1	0.887
60+	117	80.1	17	11.6	12	146	74.9	
**Gender**								
Male	88	80.7	10	9.2	11	109	55.9	0.213
Female	67	77.9	14	16.3	5	86	44.1	
**Ethnicity**								
non-Māori	131	79.4	19	11.5	15	165	84.6	0.759
Māori	23	79.3	5	17.2	1	29	14.9	
Unknown	1	100.0	0	0.0	0	1	0.5	
**Comorbidity**								
0–1	123	78.8	19	12.2	14	156	80.0	0.356
2+	32	82.1	5	12.8	2	39	20.0

**Table 4:** Participant responses to the question: Do you have confidence and trust in your GP?

Factors	Yes definitely		Yes to some extent/not at all		Doesn’t apply	Totals		
	*n* = 152	%	*n* = 40	%	*n* = 3	*N* = 195	%	p
**TDI**								
< 120 days	70	88.6	8	10.1	1	79	40.5	0.052
> 120 days	71	68.3	31	29.8	2	104	53.3	
Unknown	11	91.7	1	8.3	0	12	6.2	
**Age**								
< 60	30	61.2	18	36.7	1	49	25.1	0.004*
60+	122	83.6	22	15.1	2	146	74.9	
**Gender**								
Male	90	82.6	16	14.7	3	109	55.9	0.028*
Female	62	72.1	24	27.9	0	86	44.1	
**Ethnicity**								
non-Māori	134	81.2	29	17.6	2	165	84.6	0.104
Māori	17	58.6	11	37.9	1	29	14.9	
Unknown	1	100.0	0	0.0	0	1	0.5	
**Comorbidity**								
0–1	120	76.9	33	21.2	3	156	80.0	0.067
2+	32	82.1	7	17.9	0	39	20.0	

*p = 0.05

### Free text comments

Three themes were identified from participant free text comments: GP Interpersonal skills, technical competence and organisation of general practice care.

### Theme 1: GP interpersonal skills

The first theme identified related to the interpersonal manner of GPs, including communication, listening, showing empathy and taking symptoms seriously; skills that contribute to a positive patient-GP connection. Most participants rated their GP as ‘Very good’ or ‘Good’ in their communication:

*….GP is fantastic - he takes the time to explain everything, and is very patient (Male, age 82, stage 1, TDI<120 days)*

However, some participants voiced dissatisfaction with their GPs level of communication, which left participants feeling dismissed and poorly connected to their care:

*I had a lot of symptoms, for more than a year that I was always telling him about. I think he thought I was a hypochondriac... Around August 2017 I was very sick, vomiting and tired. I went to the GP, he ruled out the flu and said it must be another infection and left it at that (Female, age 72, stage unknown, TDI>120 days)*

*GPs vary a lot – I have had 5 different GPs - all different in their manner. Some thorough, some do not take [me] seriously. It’s important that you feel listened to - I felt like I was only being listened to by 2 out of the 5 (Female, age 67, stage unknown, TDI>120 days)*

*I had been to the GP three times in January over the pain and an obvious lump I could feel. I was getting desperate, and took my wife with me. I felt I was not being listened to (Male, age 65, stage 4, TDI>120 days)*

For some, the perception of a poor GP relationship prompted a change to a different GP or medical practice. This was the case for two participants, who felt particularly dismissed by their GPs. One described a stressful 8 month ‘fight’ to get her GP to listen and initiate a specialist referral and the other felt totally disconnected to her care:

*I had a fight with my GP- told him I would make a complaint. Begged him to send me through as urgent……felt he never examined me or listened - I was in and out quickly. I was 2 minutes late for one GP appointment and they refused me….I have since changed GP (Female, age 55, stage 3, TDI>120 days)*

*GP’s don’t seem to want to connect with you, feel rushed, didn’t want to deal with anything too complicated. Felt they are not concerned with you, felt dismissed…..the GP didn’t explain things well enough and was in ‘auto-mode’. I can't warm to her and have asked to see someone else (Female, age 58, stage 2, TDI<120 days)*

For some participants, feeling dismissed and not taken seriously by their GP directly influenced a low feeling of confidence and trust:

*[confidence]….not in the first GP, who shrugged off stomach pain as a stomach virus (Female, age 74, stage unknown, TDI>120 days)*

*I have seen my GP countless times and was told back in 2016 that I was ‘too young’ to have bowel cancer when I asked if symptoms could be the start of something like that (Male, age 41, stage unknown, TDI>120 days)*

However, some participants were more accepting of their GP’s interpersonal style, which did not affect confidence levels. One participant gave an honest description of his GP’s poor communication, yet still had total faith in his care:

*He is terrible at explaining things. I have a long standing relationship with him, and even though he has quirky weird ways, he has proven his level of care to my family multiple times – when the chips are down, you can't beat him (Male, age 76, stage 3, TDI<120 days)*

### Theme 2: technical competence

A GPs technical competence was also typically appraised by participants during appointments, and provided the second theme identified. Technical skills were often judged by the speed in which a referral was made, which for some patients, was connected to levels of confidence and trust:

*I don't have any confidence in the GP now. She was on the wrong track, had diagnosed ‘microscopic colitis’. I had been complaining about worsening symptoms for months (Female, age 52, stage 3, TDI>120 days)*

*I see different [GPs] all the time and was being monitored for low iron…..it took the Dr a long time to figure out what was wrong….GP does not have good rapport…..took too long to diagnose (Male, age 75, stage 3, TDI>120 days)*

Participants also assessed technical competence by the accuracy in which their GP reached a correct diagnosis, with many participants reporting being misdiagnosed and treated for conditions other than cancer:

*I had consulted a GP and they said if the blood was fresh it was likely to be haemorrhoids (Male, age 63, stage 3, TDI>120 days)*

*The GP diagnosed an ulcer for the abdominal pain and gave laxatives for the constipation (Female, age 73, stage unknown, TDI>120 days)*

Low confidence and feelings of dismissal were also evident in those participants who recounted being misdiagnosed in the absence of a physical examination:

*The GP misdiagnosed prostate cancer without doing any prostate cancer checks (Male, age 70, stage 2, TDI>120 days)*

*He could have done better, as soon as he knew there was blood, he should have done something sooner, despite me stating to him that it could be haemorrhoids - he never did a physical check (Female, age 86, stage 3, TDI>120 days)*

*I was pretty much going to the GP every month, and felt like I was getting nowhere….. I told the GP about the blood in my stool, but he asked whether I thought it could be piles - and never had a look himself to check…..I felt nobody was listening, I had a terrible experience….it was only in October when I begged him to send me to the hospital that I was seen (Female, age 55, stage 3, TDI>120 days)*

*The GP did some blood tests, said everything was clear but declined to view a picture I had taken of blood in the toilet bowl and did not do a physical exam…… felt like they didn't want to deal with a complicated case. I never want to go back (Female, age 71, stage 3, TDI>120 days)*

However, some participants still had confidence and trust in their GP, despite experiencing a longer diagnostic interval – especially if their GP was actively engaged in investigating symptoms, or participants could acknowledge that their own medical history was a significant contributor to diagnostic difficulty:

*One said I was ‘too young for cancer’ but still referred me, and did bloods (Female, age 31, stage 3, TDI>120 days)*

*I have a history of endometriosis, so felt their assessments were fair (Female, age 37, stage unknown, TDI>120 days)*

### Theme 3: organisation of general practice care

While clearly beyond the scope of a GPs interpersonal and technical competence, many participants commented on health system issues within general practice, suggesting that some participants do not view these as distinctly separate from the patient-GP relationship - and in fact include these factors when assessing feelings of confidence and trust in their GP. Timing of appointments was a common concern, with short appointment times resulting in participants feeling rushed and not being given enough time for their concerns to be properly heard:

*GPs are so limited with time, so they don't explain things fully….I did not feel I was being listened to. My GP only works 2 days a week, I want a GP who is available more often (Male, age 65, stage 4, TDI>120 days)*

*My GP does not like to waste his time with unnecessary conversation…..he’s a difficult bastard…….the good ones do not have time - only 10 minutes per person (Male, age 76, stage 1, TDI>120 days)*

*I changed GP - was sick of getting 10 minutes for one problem – my GP was just too blasé (Female, age 54, stage 3, TDI>120 days)*

Continuity of care was another main concern. While busy practices might offer an appointment with another GP, participants often desired to see the same GP who they felt they trusted:

*….an issue with getting to see the GP you want at my medical centre - there is a delay in getting to see who you want to see (Male, age 69, stage 1, TDI>120 days)*

*[My] GP was away on holiday, and I did not want to see another doctor in the interim. I wanted to see someone I knew (Male, age 68, stage 3, TDI>120 days)*

*I don't always see the same GP, and I would prefer to. The practice is very busy (Female, age 64, stage unknown, TDI<120 days)*

*I changed practice two years ago, due to a lack of continuity of a regular GP (Male, age 72, stage unknown, TDI<120 days)*

However, other participants were more pragmatic about having consultations with different GPs:

*They do a good job. Don't mind seeing different doctors as they have different ideas (Male, age 77, stage unknown, TDI>120 days)*

*Even if I can't get any appointment with my GP, I can see another doctor. My GP is very popular, but I don't mind seeing someone else (Male, age 67, stage unknown, TDI<120 days)*

## Discussion

We investigated perspectives of the patient-GP relationship in the context of bowel cancer detection and diagnosis by analysing the free text comments and GP ratings from recently diagnosed CRC patients. A diagnosis of cancer is a critical time for a patient, in which expectations of general practice are high. Over half of the current cohort experienced a TDI of more than 120 days. Almost 30% of participants who had a long diagnostic interval gave a low rating of confidence and trust in their GP, suggesting that for these participants, a longer TDI was one of the contributing factors to this rating. Poor interpersonal skills, such as a lack of communication or listening, and poor technical competence, including misdiagnoses and not being thoroughly examined are factors that also impact on the diagnostic experience for patients and their level of confidence and trust in the patient-GP relationship.

While it was encouraging to see many participants rating GP communication positively, several participants voiced dissatisfaction with their GPs interpersonal manner, with some participants feeling ‘desperate’ to get their GP to listen, being made to feel like a hypochondriac, or left ‘fighting’ to be taken seriously. These feelings certainly contributed to a poor overall rating of confidence and trust for some participants. Patients value having their symptoms taken seriously [1], and want to feel that their GP understands their symptoms from their perspective [22–24]. This is especially important for patients disclosing often embarrassing CRC symptoms, and for Māori patients in particular, where revealing symptoms to an (often) non-Māori practitioner may be particularly difficult [25] - especially in the light of current inequities, where Māori have a lower incidence [26] but worse CRC outcomes [27, 28], and less access to chemotherapy [29] and colonoscopy [30]. Consistent with other research [11], younger participants in the current study reported a sense of not being taken seriously and significantly more females reported low confidence in their GP. Young patients [13, 31] and females [8, 13, 32–34] are more at risk for delayed diagnosis, and the experiences of some of the participants in this study emphasize the importance of GPs not being dismissive of symptoms in these groups.

Participants also expressed dissatisfaction with the technical competence of their GP, commenting on the speed in which specialist referrals were made, often perceiving that their GP ‘took too long to diagnose’. Misdiagnoses, especially in patients who experienced a longer TDI, were commonly reported by participants, and are a significant barrier to both patients seeking further GP consultations and GPs reaching a diagnosis [35]. However, accurate diagnosis of CRC symptoms is difficult [8, 9], and GPs must interpret symptoms in the light of a number of factors, including the presence of comorbid conditions which may disguise CRC symptoms and increase time to diagnose [10, 36]. NZ GPs are also disadvantaged by less direct and slower access to colonoscopy than GPs in other countries [37], largely due to a public hospital system that is based on triage for degree of need, resulting in patients who are not likely to be seen or treated within 6 months being routinely referred back to GPs without being seen. Of concern, however, were the participant accounts of misdiagnoses in the absence of a physical examination. Low rates of physical examination prior to diagnosis have been previously reported [6, 8, 34, 38, 39], and were one of the primary sources of complaint against NZ GPs [2], so are clearly an ongoing issue in CRC diagnosis in NZ, and one that contributes to a patients overall perception of the quality of their relationship with their GP.

Organisation of general practice care, while not under direct control or responsibility of many GPs, was another prominent theme. Half of the participants who provided free text comments reported an inability to access their GP for an appointment within 24 h over the preceding 12 months. Participants also commented on appointment length, feeling that a ‘10 min slot’ was not long enough to have their issues heard. NZs standard 15 min appointment time is a funding issue, and has been raised as a point of concern by both GPs and primary care nurses [40]. Patients value GPs taking time during appointments [41], and do not like feeling rushed [23, 42], therefore taking the time within existing consult times to carefully listen may help mitigate short appointment times. Clearly this is a balancing act for GPs. Getting an appointment with a desired GP was also highly valued. Irrespective of TDI, participants expressed frustration at not being able to see the same GP, or being offered a different GP for each appointment. Poor relational continuity of care, where a lack of consistency provides patients with unpredictability and no coherence [43], is a source of patient unhappiness [9], increases time to diagnosis [13], and makes patients feel like they are being treated impersonally [42]. This is a particular issue for Māori patients, who value continuity of care [44] but do not get offered the same choice of GP appointments [45]. We suggest that further investment is needed in primary care, and that primary care organisations focus on improving continuity and patient-GP communication.

Few studies have investigated the patient-GP relationship following a CRC diagnosis from the patient’s perspective. We used a mixed methods approach to allow participant voices to be heard. Free text comments provide valuable additional data and are one way to measure a wider range of topics that might not be fully captured with a structured questionnaire [11]. However, these are not representative and so cannot be generalised to the views of all participants. Furthermore, patients with a CRC diagnosis are not typical of all cancer patients, so may experience the diagnostic pathway through general practice differently. Data collected was patient-reported, so relied on subjective memory of events and accurate recall of diagnostic dates. While interviews aimed to be conducted as close to diagnosis date as possible (at least within 12 months of diagnosis), patient recall may not have been accurate. Finally, while patient gender could be reported, GP characteristics (including age, gender, time in practice, practice size etc.) were unknown and would be important factors for inclusion in future research.

We report that long diagnostic intervals for CRC are still occurring in primary care, and that patients associate a poor diagnostic experience with deficits in the interpersonal and technical skills of their GP, and health system factors within general practice. Many of the issues reported here have been previously raised by an HDC report (2004–2013) [2]. Increased funding into primary care might help address some of these ongoing issues. While the majority of participants in the current study had confidence and trust in their GP, the diagnostic experience was extremely negative for some participants, particularly young patients, Māori, and females. Access to general practice plays a pivotal role and is particularly important to ensure equity for Māori patients.

## Data Availability

The data analysed for the current study are not publically available for ethical reasons. Anonymised data can be made available from the corresponding author on request.

## Abbreviations

NZ: New Zealand
CRC: Colorectal cancer
GP: General practitioner
MPT: The Model of Pathways to Treatment
DHB: District health board
ED: Emergency department
COBH: Changes of bowel habit
